# Standardised Ki‐67 proliferation index assessment in early‐stage laryngeal squamous cell carcinoma in relation to local control and survival after primary radiotherapy

**DOI:** 10.1111/coa.13449

**Published:** 2019-11-05

**Authors:** Emiel Kop, Geertruida H. de Bock, Maartje G. Noordhuis, Lorian Slagter‐Menkema, Bernard F. A. M. van der Laan, Johannes A. Langendijk, Ed Schuuring, Bert van der Vegt

**Affiliations:** ^1^ Department of Otorhinolaryngology University Medical Center Groningen Groningen The Netherlands; ^2^ Department of Epidemiology University of Groningen, University Medical Hospital Groningen Groningen The Netherlands; ^3^ Department of Radiotherapy University of Groningen, University Medical Hospital Groningen Groningen The Netherlands; ^4^ Department of Pathology & Medical Biology University of Groningen, University Medical Hospital Groningen Groningen The Netherlands

**Keywords:** disease‐specific survival, head and neck cancer, Ki‐67, laryngeal carcinoma, local control, proliferation, radiotherapy

## Abstract

**Objectives:**

Ambiguous results have been reported on the predictive value of the Ki‐67 proliferation index (Ki‐67 PI) regarding local control (LC) and survival after primary radiotherapy (RT) in early‐stage laryngeal squamous cell cancer (LSCC). Small study size, heterogenic inclusion, variations in immunostaining and cut‐off values are attributing factors. Our aim was to elucidate the predictive value of the Ki‐67 PI for LC and disease‐specific survival (DSS) using a well‐defined series of T1‐T2 LSCC, standardised automatic immunostaining and digital image analysis (DIA).

**Methods:**

A consecutive and well‐defined cohort of 208 patients with T1‐T2 LSCC treated with primary RT was selected. The Ki‐67 PI was determined using DIA. Mann‐Whitney *U*‐tests, logistic and Cox regression analyses were performed to assess associations between Ki‐67 PI, clinicopathological variables, LC and DSS.

**Results:**

In multivariate Cox regression analysis, poor tumour differentiation (HR 2.20; 95% CI 1.06‐4.59, *P* = .04) and alcohol use (HR 2.84, 95% CI 1.20‐6.71; *P* = .02) were independent predictors for LC. Lymph node positivity was an independent predictor for DSS (HR 3.16, 95% CI 1.16‐8.64; *P* = .03). Ki‐67 PI was not associated with LC (HR 1.59; 95% CI 0.89‐2.81; *P* = .11) or DSS (HR 0.98; 95% CI 0.57‐1.66; *P* = .97). In addition, continuous Ki‐67 PI was not associated with LC (HR 2.03; 95% CI 0.37‐11.14, *P* = .42) or DSS (HR 0.62; 95% CI 0.05‐8.28; *P* = .72).

**Conclusion:**

The Ki‐67 PI was not found to be a predictor for LC or DSS and therefore should not be incorporated in treatment‐related decision‐making for LSCC.

**Key points**

- Ambiguous results regarding the predictive value of the Ki‐67 proliferation index regarding local control and survival after primary radiotherapy have been reported in early‐stage laryngeal squamous cell cancer.
- Small study size, heterogeneous inclusion, variations in immunostaining and cut‐off values are factors attributing to these contradictory results.
- We used a well‐defined series of T1‐T2 laryngeal tumours treated with radiotherapy, standardised automatic immunostaining and automatic digital scoring.
- Standardised and automated staining minimises variable staining intensity and improves reproducibility. Automated digital scoring eliminates interobserver variability.
- The Ki‐67 proliferation index was not a predictor for local control or disease‐specific survival and therefore should not be incorporated in treatment‐related decision‐making for early‐stage laryngeal squamous cell cancer.

## 1 INTRODUCTION

Over the years, many studies have been conducted to identify prognostic and predictive markers for head and neck squamous cell carcinoma (HNSCC).^1^ To date, prognostic markers such as age, TNM‐stage and histological type determine decision‐making regarding the most optimal treatment strategy. In oncogenesis, cell proliferation is one of the most essential biological processes and may therefore be a strong predictive and prognostic marker.^2^ Ki‐67 is a nuclear marker that is present in all phases of the cell cycle but absent in resting cells (G0 phase).^3^ Therefore, Ki‐67 is an ideal marker to quantify the relative amount of proliferative neoplastic cells within tumour tissue, defined as the Ki‐67 proliferation index (Ki‐67 PI). However, the results of earlier studies investigating the relationship between the Ki‐67 PI, local control (LC) and survival after primary RT in laryngeal squamous cell cancer (LSCC) are not unambiguous, as shown in Table 1.^4^, ^5^, ^6^, ^7^, ^8^, ^9^, ^10^, ^11^, ^12^, ^13^, ^14^ Possible explanations for these differences are variations in patient group factors, immunostaining and scoring‐related factors.^15^, ^16^, ^17^

**Table 1:** Patients and disease characteristics related to local recurrence after radiotherapy

First author year	Method	Cut‐off	N	Stage	Side	Local control, definition	Treatment	Univariate HR/OR (95% CI)
Kropveld et al^4^ 1998	IHC	Continuous	36	T2N0‐2	Larynx	LR	RT	↑
Sakata et al^5^ 2000	IHC	≥50%	130 51 79	T1‐2N0	Glottic	LR	RT & ART	↓2.66 (1.17‐6.08)^a^ =1.32 (0.40‐4.38)^a^ ↓5.11 (1.53‐17.04)^a^
Motamed et al^6^ 2001	IHC	Continuous	28	T1aN0	Glottic	Radioresistance, n.s.	n.s.	=
Condon et al^7^ 2002	IHC	>20%	21	T1‐2N0	Glottic	LR < 12 mo	RT	=1.94 (0.32‐11.8)^a^
Cho et al^8^ 2004	IHC‐TMA	≥10%	123	T1‐2N0	Larynx	Time to LR < 5 y	RT	=0.47 (0.18 −1.23)^a^
Ahmed et al^9^ 2008	IHC	>10% Continuous	24	T1‐2	Glottic	LR or persistence	RT	↑, =
Rafferty et al^10^ 2008	IHC	>50% Continuous	50	T2N0	Larynx	LR	HRT	↑, =
Wildeman et a^11^ 2009	IHC‐TMA	Continuous	59	T1‐3N0‐3	Larynx	LR < 2 y	RT	=0.71 (0.44‐1.15)
Nichols et al^12^ 2012	IHC	>10%	75	T1‐2	Glottic	Time to LR	RT	↓3.37 (1.14‐9.86)
Rademakers et al^13^ 2015	IHC	>10%	128	T2‐4N0‐+	Larynx	Time to LR	ART	=
Kwon et al^14^ 2015	IHC	>50%	42	T1‐2	Larynx	Residual tumour < 6 mo	RT	=2.16 (0.40‐11.80)
Kop et al 2018 (this study)	IHC	≥50%	208	T1‐2N0‐3	Larynx	LR < 2 y	RT	=1.58 (0.89‐2.79)

Abbreviations: ART, accelerated radiotherapy; CI, confidence interval; HR, hazard ratio; HRT, hypofractionated radiotherapy; IHC, immunohistochemistry; LR, local recurrence; mo, months; n.s., not specified; OR, odds ratio; RT, conventional radiotherapy; TMA, tissue microarray; yrs, years.

a (Subgroup) analysis performed by authors of this article.

^↓^ High Ki‐67 associated with poor local control.

^↑^ High Ki‐67 associated with good local control.

^=^ No relation with local control.

The aim of this study was to assess the value of Ki‐67 PI in predicting LC and disease‐specific survival (DSS) after primary RT in a well‐defined consecutive series of patients with early‐stage (T1‐T2) LSCC. By using standardised and automated immunohistochemistry along with digital image analysis (DIA) to assess the Ki‐67 PI, we reduced staining and scoring variability.

## 2 PATIENTS AND METHODS

### 2.1 Patients

Patients treated for LSCC at our institution are included in a database by the Netherlands Cancer Registry (NCR) by using the results of the nationwide network and registry of histo‐ and cytopathology in the Netherlands (PALGA). Retrospectively, data from the hospital consisting of date of birth, sex, tumour site, TNM status, tumour classification and therapy modality are collected.^18^, ^19^ From this database, a consecutive series of patients was included in the current study who were (a) diagnosed between 1990 and 2012; (b) with a primary T1‐T2 biopsy‐proven LSCC; (c) had received and completed primary RT with curative intent; (d) had a minimum follow‐up of 5 years (if not deceased); and (e) had biopsy tissue available in the biobank at the Department of Pathology of our institution. Patients who had a coincidental lung carcinoma, multiple HNSCC or previous radiation or surgery of the head and neck region were excluded. Additional clinical, histopathological and follow‐up data were retrospectively collected. Initially, 317 patients could be included. Sufficient biopsy material was available in 238 cases. After Haematoxylin and Eosin (HE) staining and reviewing by a head & neck pathologist, 30 biopsy specimens were additionally excluded because of insufficient invasive tumour tissue within the biopsy material. This resulted in a cohort of 208 patients. The majority of included patients were used in previous studies regarding the evaluation of other biomarkers.^20^, ^21^, ^22^

### 2.2 Ethical considerations

According to the Central Committee on Research involving Human Subjects (CCMO), this type of study did not require approval from an ethics committee in the Netherlands. This study was approved by the Privacy Review Board of the NCR by following “The Code of Conduct for the Use of Data in Health Research” of the CCMO.^23^

### 2.3 Treatment

**Figure 1:**
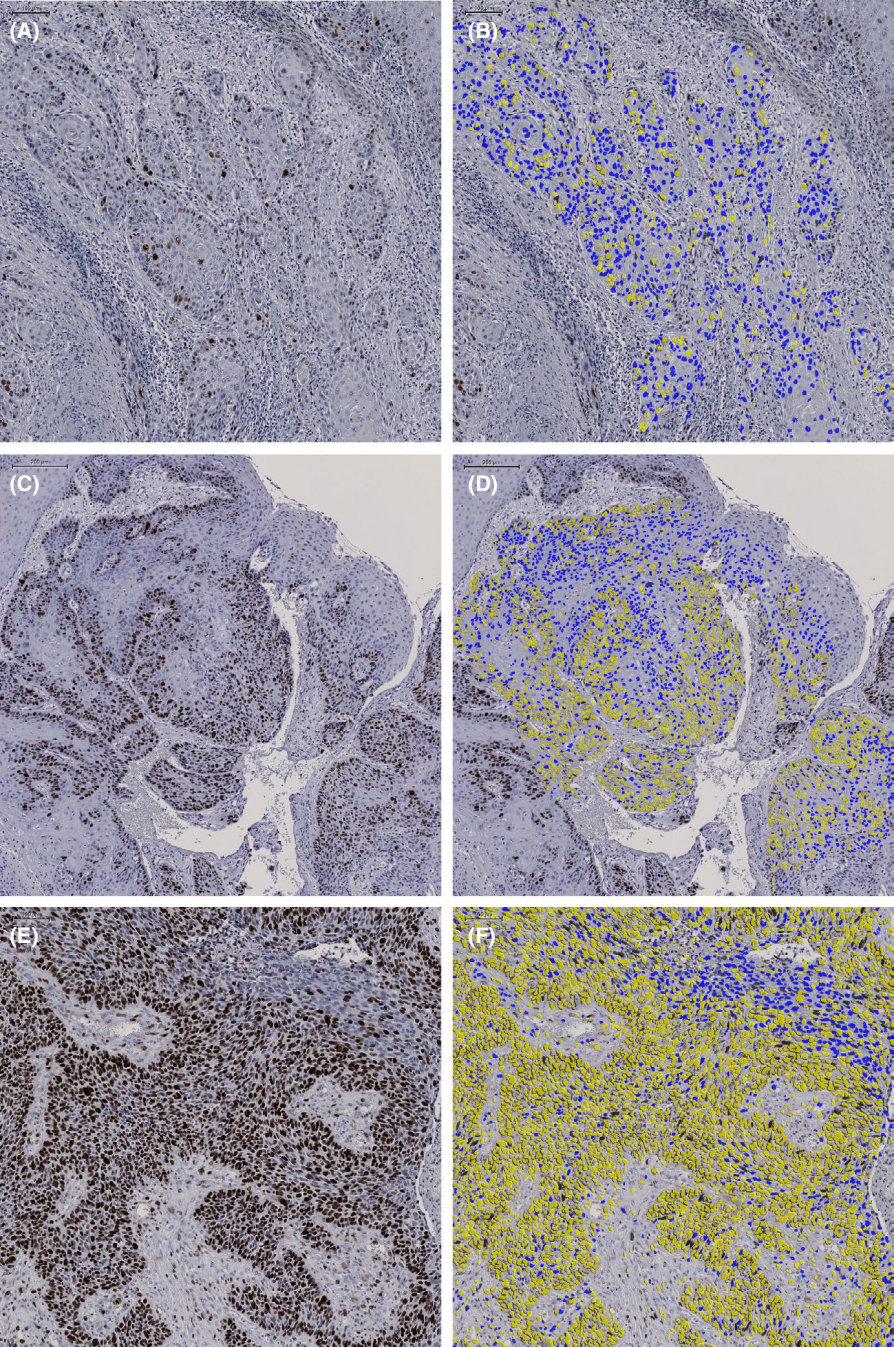
Examples of nucleus detection with positive (green) and negative (blue) marked tumour cells for nuclear Ki‐67 staining. Ki‐67 expression: (A, B) low (C, D) intermediate (E, F) high

All patients were treated by a multidisciplinary head & neck team. Patients received primary RT with curative intent using 6MV linear accelerator equipment as previously described.^20^, ^21^, ^22^ In short, T1 tumours received 2 Gy fractions five times weekly with a total dose of 66 Gy. T2 tumours were treated with six fractions weekly to a total dose of 70 Gy. In case of lymph node metastasis, a total dose of 46 Gy was electively delivered to the primary planning target volume together with an additional boost of 70 Gy to the primary tumour and pathologic lymph nodes. From the year 2000 onwards, planning of field arrangements was performed by using contrast‐enhanced computed tomography (CT). Before 2000, this was calculated by direct simulation (Figure 1).

### 2.4 Follow‐up

All patients had standardised follow‐up after completing RT in accordance with the Dutch Working Party on Head and Neck Tumours (NWHHT) guidelines.^24^ For the first 2 years, the otorhinolaryngology and radiotherapy department alternately performed physical examination with laryngoscopy every 3 months. After 2 years, this was alternately performed every 6 months up till 5 years after completing radiation treatment. Patients were discharged from follow‐up after 5 years if no evidence of disease was found.

### 2.5 Immunohistochemistry

All tumour material was formalin‐fixed and paraffin‐embedded. Using a standard microtome, 3 µm sections were cut from the tumour paraffin blocks. Immunohistochemistry for Ki‐67 (CONFIRM^®^ anti‐Ki‐67 [30‐9] Rabbit Monoclonal Primary Antibody, Ventana Medical Systems) was performed using the automated Benchmark^®^ platform (Ventana Medical Systems) according to the manufacturer's recommendations and protocol. The antibody was pre‐diluted by the supplier.

### 2.6 Evaluation of immunohistochemical staining

A whole tumour slide was analysed in order to reduce sampling error. All glass slides digitized using the Hamamatsu Nanozoomer HT 2.0 (Hamamatsu Photonics KK, 325‐6). For semi‐automated DIA, Definiens Tissue Studio 3.6 (Definiens AG) was used. Images were processed in one batch automatically for identification and calculation of nuclear staining. Twenty random cases were selected and manually counted by the head & neck pathologist to validate the image analysis algorithm. All slides contained at least 500 countable cells (median 7308, range 509‐121.847).

### 2.7 Definitions

LC was defined as local tumour recurrence at the primary tumour site within 2 years after RT and was calculated from the date of diagnosis until the date of recurrence. After this period of 2 years, any local recurrence was defined as a second primary tumour. DSS was defined as the date of diagnosis until the date of death by disease or last date of follow‐up within 5 years.

In the analyses, Ki‐67 PI was considered both as a continuous and a dichotomous variable. For dichotimisation, the cut‐off value for high vs low Ki‐67 PI was set to 50%, which was defined by the median Ki‐67 expression in our cohort. In addition, we also tried to compare our data with previously published studies, which used cut‐off values of 10% and 20%.^7^, ^8^, ^9^, ^12^, ^13^

Alcohol use was defined as drinking one or more units per day either in the past or at date of diagnosis. The same was applied for tobacco use with smoking one or more cigarettes or sigars per day.

### 2.8 Statistical analysis

Patients were dichotomised based on their Ki‐67 PI, and for correlations between patient and tumour characteristics, univariate logistic regression was used. In this way, odds ratios (OR) and 95% confidence intervals (95% CI) were estimated for a high Ki‐67 PI. When the Ki‐67 PI was considered as a continuous variable, Mann‐Whitney *U*‐tests were performed. The correlation between the Ki‐67 PI and clinicopathological characteristics was evaluated for LC and DSS by using univariate Cox regression analyses estimating hazard ratios (HR) and 95% CI. All statistical tests were two‐sided and a *P*‐value ≤ .05 was considered to be statistically significant. For multivariate analysis, factors with a *P*‐value of > .15 were excluded in a stepwise manner; factors with a *P*‐value of ≤ .05 were included in the final step. Statistical analyses were performed using SPSS (IBM Corp. Released 2015. IBM spss Statistics for Windows, Version 23.0: IBM Corp).

## 3 RESULTS

Patient and tumour characteristics are described in Table 2. The majority of tumours were of glottic origin, had a T2 status, did not have lymph node metastasis and were moderately differentiated. Most patients were male and median age was 64.4 years.

**Table 2:** Pre‐treatment Clinical and Tumour Characteristics (N (%), unless specified otherwise) Characteristics

	Total n = 208
Age, years	
Median (range)	64.4 (33‐96)
<64	110 (52.9)
≥64	98 (47.1)
Gender	
Female	22 (10.6)
Male	186 (89.4)
Alcohol use	
No	57 (27.4)
Yes (≥1 units/d)	135 (64.9)
Unknown	16 (7.7)
Tobacco use	
No	26 (12.5)
Yes (≥1 cig/d)	176 (84.6)
Unknown	6 (2.9)
Subsite	
Glottic	146 (70.2)
Supragottic	62 (29.8)
cT‐status	
T1	84 (40.4)
T2	124 (59.6)
cN‐status	
N0	188 (90.4)
N+	20 (9.6)
1	13
2	6
3	1
Differentiation	
Well	26 (12.5)
Moderate	160 (76.9)
Poor	22 (10.6)

At the date of analysis, median follow‐up time was 65.2 months (range 4‐236). Local recurrence occurred in 48 patients, of which 40 patients underwent a total laryngectomy with or without additional neck dissection. Eight patients received palliative treatment. The median time to local recurrence was 9.6 months (range 5‐21). After 5 years, 152 patients were still alive, 21 patients died from disease, 26 patients died unrelated to disease and nine patients died from unknown causes.

Specific nuclear Ki‐67 staining was present and DIA could be performed in all cases (Figure 1). Median Ki‐67 PI was 49% (range 4%‐89%). As this approached the 50% cut‐off used in other studies, we also used a 50% cut‐off between high and low Ki‐67 PI for comparability. Using a 10% and 20% cut‐off, a high Ki‐67 PI was found in 207 cases (99.5%) and 199 cases (95.7%), respectively. As the low Ki‐67 PI group was too small for both cut‐offs (one and nine cases respectively), no further statistical analyses were performed using these cut‐offs.

In the univariate regression analysis using a 50% cut‐off for the Ki‐67 PI, no significant associations between clinicopathological variables and Ki‐67 PI were found (Table 3). When treated as a continuous variable, no significant associations between Ki‐67 PI and the evaluated variables were found (Table 3).

**Table 3:** Patient and tumour characteristics related to high (≥50%) Ki‐67 PI (univariate logistic regression analyses) and continuous Ki‐67 values (Mann‐Whitney *U*‐Test)

Characteristics	Total	High Ki‐67 PI		Continuous Ki‐67 PI
		OR (95% CI)	*P* value	*P* value^a^
Age (continuous)	208	1.01 (0.99‐1.04)	.32	n/a
Age, years				
<64	98	1		
≥64	110	1.27 (0.73‐2.20)	.39	.74
Gender				
Female	22	1		
Male	186	0.75 (0.31‐1.82)	.52	.20
Alcohol				
No	57	1		
Yes (≥1 units/d)	135	1.15 (0.62‐2.15)	.65	.86
Tobacco use				
No	26	1		
Yes (≥1 cig/d)	176	0.91 (0.40‐2.08)	.83	.44
Subsite				
Glottic	146	1		
Supraglottic	62	1.77 (0.97‐3.24)	.62	.10
cT‐status				
T1	84	1		
T2	124	1.03 (0.59‐1.80)	.93	.26
cN‐status				
N0	188	1		
N+	20	2.16 (0.82‐5.65)	.12	.12
Differentiation				
Well/moderate	186	1		
Poor	22	1.34 (0.55‐3.25)	.52	.66

Abbreviations: 95% CI, 95% Confidence Interval; Ki‐67 PI, Ki‐67 proliferation index; n/a, not applicable; OR, Odds Ratio.

a Mann‐Whitney *U* Test.

A significant negative association between LC and poor tumour differentiation (HR 2.18; 95% CI 1.06‐4.50, *P* = .04), alcohol use (HR 2.94, 95% CI 1.24‐6.95; *P* = .01) and tobacco use (HR 7.59; 95% CI 1.05‐55.02, *P* = .045) was found. In stepwise multivariate Cox regression analysis, alcohol use (HR 2.84, 95% CI 1.20‐6.71; *P* = .02) and poor differentiation (HR 2.20; 95% CI 1.06‐4.59, *P* = .04) were independent predictors for worse LC. No associations between high Ki‐67 PI and LC (HR 1.59; 95% CI 0.89‐2.81; *P* = .11) or KI67 PI as a continuous variable and LC (HR 2.03; 95% CI 0.37‐11.14; *P* = .42) were found (Figure S1A, Table 4).

**Table 4:** Patient and tumour characteristics related to local control (univariate and multivariate cox regression analyses)

Characteristics	Total	LC (univariate)		LC (multivariate)	
		HR (95% CI)	*P* value	HR (95% CI)	*P* value
Age (continuous)	208	0.99 (0.97‐1.02)	.71	a	
Gender					
Female	22	1			
Male	186	1.29 (0.46‐3.60)	.62	a	
Alcohol use					
No	57	**1**			
Yes (≥1 units/d)	135	**2.94 (1.24‐6.95)**	**.01**	**2.84 (1.20‐6.71)**	**.02**
Tobacco use					
No	26	**1**			
Yes (≥1 cig/d)	176	**7.59 (1.05‐55.02)**	**.045**	6.78 (0.93‐49.25)	.06
Subsite					
Glottic	146	1			
Supraglottic	62	1.21 (0.66‐2.20)	.54	a	
cT‐status					
T1	84	1			
T2	124	1.43 (0.78‐2.61)	.24	a	
cN‐status					
N0	188	1			
N+	20	1.68 (0.75‐3.75)	.20	a	
Differentiation					
Well/moderate	186	**1**			
Poor	22	**2.18 (1.06‐4.50)**	**.04**	**2.20 (1.06‐4.59)**	**.04**
Ki‐67 PI					
Low	108	1			
High	100	1.59 (0.89‐2.81)	.12	b	
Ki‐67 PI (continuous)	208	2.03 (0.37‐11.14)	.42	a	

Abbreviations: 95% CI, 95% Confidence Interval; HR, Hazard Ratio; Ki‐67 PI, Ki‐67 proliferation index; LC, Local control.

Significant results are shown in bold.

a Not included in multivariate analysis.

b Not included in final step of multivariate analysis.

In univariate and stepwise multivariate Cox regression analysis, a significant negative association was found between lymph node positivity and DSS (HR 3.16, 95% CI 1.16‐8.64; *P* = .03). No associations were found between Ki‐67 PI and DSS (HR 0.98; 95% CI 0.57‐1.66; *P* = .97) or KI67 PI as a continuous variable and DSS (HR 0.62; 95% CI 0.05‐8.28; *P* = .72) (Figure S1B, Table 5).

**Table 5:** Patient and tumour characteristics related to disease‐specific survival (univariate cox regression analyses)

Characteristics	DSS		
	Total	HR (95% CI)	*P* value
Age (continuous)	208	0.97 (0.93‐1.01)	.16^a^
Gender			
Female	22	1	
Male	186	1.16 (0.27‐4.97)	.84^a^
Alcohol use			
No	57	1	
Yes (≥1 units/d)	135	2.51 (0.74‐8.57)	.14^b^
Tobacco use			
No	26	1	
Yes (≥1 cig/d)	176	3.18 (0.43‐23.71)	.26^a^
Subsite			
Glottic	146	1	
Supraglottic	62	1.49 (0.62‐3.58)	.38^a^
cT‐status			
T1	84	1	
T2	124	0.93 (0.39‐2.21)	.87^a^
cN‐status			
N0	188	**1**	
N+	20	**3.16 (1.16‐8.64)**	**.03**
Differentiation			
Well/moderate	186	1	
Poor	22	2.49 (0.91‐6.79)	.08^b^
Ki‐67 PI			
Low	108	1	
High	100	0.99 (0.42‐2.32)	.97^a^
Ki‐67 PI (continuous)	208	0.62 (0.05‐8.28)	.72^a^

Abbreviations: 95% CI, 95% Confidence Interval; DSS, Disease‐Specific Survival; HR, Hazard Ratio; Ki‐67 PI, Ki‐67 proliferation index.

Significant results are shown in bold.

a Not included in multivariate analysis.

b Not included in final step of multivariate analysis.

## 4 DISCUSSION

In a well‐defined series of patients diagnosed with T1‐T2 LSCC and treated with primary RT, Ki‐67 PI was determined using standardised automated immunohistochemistry and DIA. No statistically significant associations between high (≥50%) or continuous Ki‐67 PI and clinicopathological characteristics, LC or DSS were found.

From the eleven previously conducted studies, 15 (sub)analyses were reported or could be calculated using the data and cut‐off values provided in the papers (Table 1). Of those, nine did not find a significant association between Ki‐67 PI and LC after RT.^6^, ^7^, ^8^, ^9^, ^10^, ^11^, ^13^, ^14^ Two subgroup analyses in one study showed a negative association between high Ki‐67 and LC in both a cohort treated with accelerated RT (ART) and in a combined cohort treated with either ART or conventional RT (HR 2.66; 95% CI 1.17‐6.08 and HR 5.11; 95% CI 1.53‐17.06 respectively).^5^ Nichols et al found a worse local, regional or distant control in patients with high Ki‐67 tumours.^12^ Three studies showed a significant positive association between high Ki‐67 and LC after RT using continuous values, and one study showed a positive association using a 50% cut‐off (no HR or 95% CI was given or could be calculated).^4^, ^9^, ^10^ However, selection bias may have influenced the outcome of these studies as in one of the studies 36 patients were randomly selected from a larger cohort of 128 patients,^9^ another study included only 24 patients with a glottic carcinoma involving the anterior commissure in a 10‐year period. The study of Rafferty et al only describes 50 patients from a prospective database, which included patients since 1960.^15^ Moreover, no multivariate analyses to correct for possible confounding factors were conducted to verify their significant associations in univariate analyses. The results of the current study are in line with results of earlier studies that included larger study groups. Cho et al concluded that Ki‐67 was not predictive for LC after primary RT treatment in a series of 123 T1‐T2N0 LSCC.^8^ A similar conclusion was drawn by Rademakers et al who also used DIA to assess Ki‐67 in 128 patients.^13^

From five subanalyses of the four studies that assessed the association between Ki‐67 and survival, none found a difference in OS,^5^, ^8^ DSS^5^, ^13^ or survival (not otherwise specified).^10^ In one paper, worse regional control and metastasis‐free survival were reported.^13^ A confounder for this result might be the inclusion of advanced LSCC, which has a much higher tendency to metastasise (regionally). Our study only consisted of early‐stage LSCC. The role of Ki‐67 in advanced tumours could be the subject of a follow‐up study.

Consensus on Ki‐67 staining protocols, Ki‐67 antibodies and scoring methods is still lacking. The published cut‐offs for high vs low Ki‐67 PI varied between 10%, 20% and 50%, along with continuous values. We believe tumour markers without pre‐set cut‐off value (ie continuous values) are deemed less fit as diagnostic biomarker for decision‐making regarding different therapeutic modalities.

Various definitions regarding LC after radiotherapy make it even more difficult to compare study results. A global definition for LC is needed in order to have better comparability across studies. Despite this lack of consensus, this seems not to be explanatory for the different outcomes.

In our cohort, we found a relatively high Ki‐67 PI compared with other studies. One of the explanations might be that we used marked HE slides to accurately determine and select neoplastic regions within the digitised Ki‐67 slide. Our digital image algorithm solely selected neoplastic cells and excluded non‐tumour cells resulting in an accurate calculation of the Ki‐67 PI. Rademakers et al who also used DIA on whole tumour section slides do not explicitly state they adjusted scoring for non‐neoplastic regions; which could have led to a lower ratio of Ki‐67 positive cells.^13^ Also, intratumour heterogeneity may lead to lower Ki‐67 PI if the incorrect region within the tumour is counted. Others studies predominately used manual counting. Most studies, if reported, counted smaller regions of the whole tumour, which could lead to selection bias by sampling error and interobserver variability.^5^, ^6^, ^8^, ^9^, ^10^

For breast carcinoma, Dowsett et al recommend counting at least 500‐1000 cells in order to compensate for intratumour proliferation heterogeneity.^15^ With our automated analysis, a median of 7308 cells (ranging 509‐121.847) was counted in a standardised, fast, reliable and reproducible manner. We previously validated the use of DIA for Ki‐67 in breast carcinoma and found a high interobserver agreement between manual and automated Ki‐67 scoring.^25^

Manual immunohistochemical staining is a time‐consuming process and leads to variable staining intensity. Interlaboratory variety is clearly illustrated in the study of Polley et al, were they investigated the interlaboratory reproducibility for Ki‐67 staining in breast cancer cases among eight North American and European laboratories.^26^ A moderate reproducibility across the laboratories was found when they used their own scoring methodology on sections stained in a central laboratory. This reproducibility declined even further when both staining and scoring were done locally. By using a standardised and automated staining platform with a pre‐diluted antibody by the supplier, we minimised this problem and improved reproducibility, enabling future interlaboratory comparison.

## 5 CONCLUSION

In this well‐defined consecutive series of T1‐T2 LSCC treated with primary RT, the clinicopathological characteristics alcohol use and poor tumour differentiation were independent predictors for worse LC. Lymph node positivity was a negative predictor for DSS. The Ki‐67 PI however did not predict outcome regarding LC or DSS after treatment. Therefore, the Ki‐67 PI should not be incorporated in treatment‐related decision‐making for LSCC.

## CONFLICT OF INTEREST

None to declare.

## Data Availability

The data that support the findings of this study are available from the corresponding author upon reasonable request.
