# Supplementary material for: Standardised Ki‐67 proliferation index assessment in early‐stage laryngeal squamous cell carcinoma in relation to local control and survival after primary radiotherapy
Source: Clin Otolaryngol. 2019 Nov 5;45(1):12–20. doi: 10.1111/coa.13449 (PMC6973088; doi:10.1111/coa.13449)
Supplement: Supplementary file 1 [file COA-45-12-s001.docx]

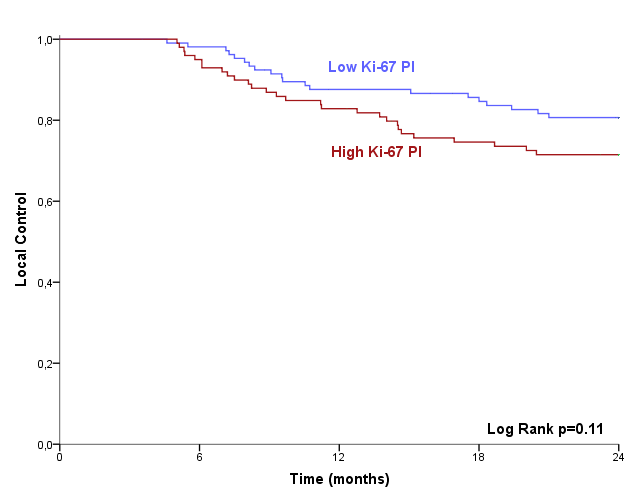

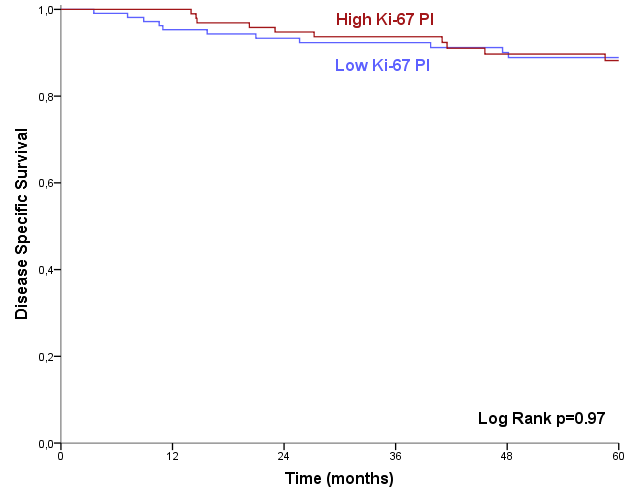


**B**

**A**

*Numbers at risk*

*Low 108 104 90 85 80 108 100 93 83 78 74*

*High 100 94 82 72 68 100 98 89 77 65 59*

**Figure S2.** (A) Local control and (B) Disease specific survival as function of low vs high Ki-67 PI in early laryngeal squamous cell carcinoma
